# Inhibitory NKG2A^+^ and absent activating NKG2C^+^ NK cell responses are associated with the development of EBV^+^ lymphomas

**DOI:** 10.3389/fimmu.2023.1183788

**Published:** 2023-06-22

**Authors:** Hannes Vietzen, Philipp B. Staber, Sarah M. Berger, Philippe L. Furlano, Laura M. Kühner, Simone Lubowitzki, Alexander Pichler, Robert Strassl, Jan J. Cornelissen, Elisabeth Puchhammer-Stöckl

**Affiliations:** ^1^ Center for Virology, Medical University of Vienna, Vienna, Austria; ^2^ Department of Medicine I, Division of Hematology and Hemostaseology, Medical University of Vienna, Vienna, Austria; ^3^ Division of Clinical Virology, Medical University of Vienna, Vienna, Austria; ^4^ Department of Hematology, Erasmus University Medical Center, Rotterdam, Netherlands

**Keywords:** EBV - Epstein-Barr virus, NK cells, LMP-1, EBV+ lymphoma, immune evasion, HLA-E polymorphism, NKG2A, NKG2C NK cells

## Abstract

Epstein-Barr virus (EBV) is a ubiquitous herpesvirus, which infects over 90% of the adult human population worldwide. After primary infections, EBV is recurrently reactivating in most adult individuals. It is, however, unclear, why these EBV reactivations progress to EBV^+^ Hodgkin (EBV^+^HL) or non-Hodgkin lymphomas (EBV^+^nHL) only in a minority of EBV-infected individuals. The EBV LMP-1 protein encodes for a highly polymorphic peptide, which upregulates the immunomodulatory HLA-E in EBV-infected cells, thereby stimulating the inhibitory NKG2A-, but also the activating NKG2C-receptor on natural killer (NK) cells. Using a genetic-association approach and functional NK cell analyses, we now investigated, whether these HLA-E-restricted immune responses impact the development of EBV^+^HL and EBV^+^nHL. Therefore, we recruited a study cohort of 63 EBV^+^HL and EBV^+^nHL patients and 192 controls with confirmed EBV reactivations, but without lymphomas. Here, we demonstrate that in EBV^+^ lymphoma patients exclusively the high-affine *LMP-1 GGDPHLPTL* peptide variant-encoding EBV-strains reactivate. In EBV^+^HL and EBV^+^nHL patients, the high-expressing HLA-E*0103/0103 genetic variant was significantly overrepresented. Combined, the LMP-1 *GGDPHLPTL* and HLA-E*0103/0103 variants efficiently inhibited NKG2A+ NK cells, thereby facilitating the *in vitro* spread of EBV-infected tumor cells. In addition, EBV^+^HL and EBV^+^nHL patients, showed impaired pro-inflammatory NKG2C^+^ NK cell responses, which accelerated the *in vitro* EBV-infected tumor cells spread. In contrast, the blocking of NKG2A by monoclonal antibodies (Monalizumab) resulted in efficient control of EBV-infected tumor cell growth, especially by NKG2A^+^NKG2C^+^ NK cells. Thus, the HLA-E/*LMP-1/*NKG2A pathway and individual NKG2C^+^ NK cell responses are associated with the progression toward EBV^+^ lymphomas.

## Introduction

Epstein-Barr virus (EBV) is a ubiquitous herpesvirus, which infects over 90% of the adult human population worldwide (1). After primary infection, EBV establishes a life-long persistent infection in memory B cells, from which sporadic reactivations may occur. EBV reactivations in immunocompetent individuals are often either asymptomatic or result in mild diseases, characterized by unspecific symptoms such as fever or fatigue.

EBV reactivations are, however, also associated with the development of malignant EBV-associated diseases, resulting worldwide in >137,900 annual deaths (1). The diffuse large B cell lymphoma (DLBCL) and peripheral T cell lymphoma (PTCL) are frequently occurring types of high-grade non-Hodgkin lymphomas (nHL). About 10% of all DLBCL and 21% of all PTCL, mainly of the *not otherwise specified* subtypes, are EBV-DNA-positive and express EBV-encoded proteins of the latency II or III viral gene expression profile (2).

Among all Hodgkin lymphoma (HL) cases, about 40%, mainly of the classical (cHL) - nodular sclerosis (NSHD) subtype are associated with EBV. EBV^+^HL are hallmarked by the presence of clonal EBV genomes and EBV-encoded proteins of the latency type II in the HL-defining Hodgkin Reed-Sternberg (HRS) tumor cells. EBV^+^HRS cells originate from germinal center B-lymphocytes, which are surrounded by an inflammatory infiltrate consisting of, among others, natural killer (NK) cells. The presence of these cytotoxic cells has, however, a minor impact on the on prognosis of EBV^+^HL, suggesting an efficient immune evasion of EBV^+^HRS cells (3).

As malignant EBV-associated diseases only occur in a fraction of EBV seropositive patients, it was hypothesized that there are distinct, individually determined factors in the infecting EBV-strains as well as human EBV-specific immune responses, that may control EBV replication and eliminate EBV infected and transformed cells.

The EBV-specific immune responses are hallmarked by potent cytotoxic CD8^+^ T cell and NK cell responses (4). EBV evolved, however, several immune evasion strategies to efficiently escape these highly cytotoxic EBV-specific immune responses. The EBV *LMP-1* gene, which is expressed during the latency type II and type III, commonly found in EBV^+^nHL and EBV^+^HL patients, encode for a highly polymorphic peptide, which stabilizes the non-classical HLA molecule HLA-E on the surface of latently EBV-infected cells (5). HLA-E is highly conserved in European populations and only two allelic variants, the high-expressing HLA-E*0103 and the low-expressing HLA-E*0101 are prevalent (6).

HLA-E further binds to the inhibitory NKG2A/CD94 as well as the activating NKG2C/CD94 receptor complexes, which are expressed on distinct NK cell subsets. By their HLA-E-stabilizing peptides, EBV infections elicit the expansion of NKG2A^+^ NK cells; a NK cell subset, which respond to EBV-infected cells by the secretion of pro-inflammatory cytokines and cellular cytotoxicity (7, 8).

Besides EBV infections, also human cytomegalovirus (HCMV) infections lead to an imprint on the human NK cell repertoire. In contrast to EBV, HCMV-infections result in the expansion of pro-inflammatory NKG2C^+^ NK cells (9). It was shown that the *KLRC2* gene, encoding for the NKG2C receptor, is homozygously and heterozygously deleted in about 4% and 32.4% of the European population, respectively (10). Homozygous and heterozygous *KLRC2* deletion is linked to decrease or even absent expression of the NKG2C, which severely impairs the activation of NKG2C^+^ NK cells.

In the present study, we hypothesized that a potent EBV *LMP-1*-mediated inhibition of NKG2A^+^ and an absent activation of pro-inflammatory NKG2C^+^ NK cells contribute to the immune evasion of EBV-infected cells and the development of EBV-associated lymphomas. By combining genetic association approaches with functional *in vitro* NK cell assays, we could demonstrate that the inhibition of NKG2A^+^ and the absence of NKG2C^+^ NK cell responses are significantly associated with the development of EBV^+^ lymphomas.

## Subjects and methods

### Study cohort

In our study, a total of 255 patients were included. Of those, 25 had confirmed EBV^+^ cHL of the NSHD subtype (EBV^+^HL), 22 had EBV^+^ diffuse large B-cell lymphoma, *not otherwise specified* (EBV^+^DLBCL) and 16 had EBV^+^ peripheral T cell lymphoma, *not otherwise specified* (EBV+PTCL). EBV^+^HL, EBV^+^DLBC and EBV^+^PTCL were diagnosed and classified according to recently published WHO guidelines (11), using histological and/or cytological findings. All lymphoma patients had a detectable EBV-viremia (>200 copies/mL plasma), EBV-VCA- and EBNA-specific IgG, but non-detectable VCA-IgM-specific IgM antibodies.

We further included 96 individuals with symptomatic EBV reactivations. These patients were tested for EBV, due to a fever of unknown origin or other unspecific symptoms. In all patients, EBV-DNA, as well as EBV-EBNA- and EBV-VCA-specific IgG antibodies were detectable. In 12 of these patients (12.5%), EBV-VCA-specific IgM titers were detectable. In all patients, no other infections than EBV were detected and none of the patients had any malignant disease within a 7-year follow-up after the study inclusion. Symptomatic EBV-infected individuals were matched to the EBV^+^HL and EBV^+^nHL cohorts in regard of age and gender using case-control matching (SPSS 25).

From each patient, one plasma sample was available: From EBV^+^HL and EBV^+^nHL patients during the EBV-viremic phase, immediately (0-9 days) after the first disease diagnosis. From symptomatic EBV-infected individuals, plasma samples were available, which were sent to the Center for Virology, Medical University of Vienna for routine EBV-diagnosis.

Furthermore, we included 96 EBV-EBNA- and EBV-VCA-specific IgG positive voluntary blood donors, who had, in spite of detectable EBV-DNA no symptoms related to symptomatic EBV reactivations. From these, 4 patients (4.2%) had EBV-VCA-specific IgM antibodies. From all asymptomatic EBV-infected individuals, no information about their age and gender were available.

For the functional assays, we also included PBMCs from additional 12 healthy, HCMV- and EBV-seropositive voluntary blood donors.

### EBV-detection and serology

Viral DNA was isolated from plasma samples using NucliSens EasyMag extractor (bioMérieux). Nucleic acids were eluted in 50 μl nuclease-free H_2_O. EBV-DNA was detected and quantified by TaqMan assays using recently published protocols (12). HCMV-specific IgG, EBV VCA-specific IgM, EBNA-specific IgG and VCA-specific IgG antibodies were detected and quantified by ELISA (all: Euroimmune).

### *LMP-1*, HLA-E and *KLRC2* genotyping

Genomic and viral DNA was isolated from 200µL plasma using the NucliSens EasyMag extractor. Nucleic acids were eluted in 50 μl nuclease-free H_2_O. *KLRC2* ^wt/del^ variants were determined by touchdown-PCR as recently described (13). HLA-E genotyping was performed using a recently published TaqMan assay and HLA-E*0101- and HLA-E*0103-specific probes (14, 15). *LMP-1* variants were determined by nested PCR, followed by Sanger-Sequencing, as described before (5). DNA sequences were translated into protein sequences using the Expasy tool, developed by the Swiss-Prot group and supported by the SIB Swiss Institute of Bioinformatics (https://web.expasy.org/translate/).

### Isolation of primary cells

Peripheral blood mononuclear cells (PBMCs) from 12 voluntary and healthy HCMV- and EBV-seropositive blood donors, were isolated from buffy-coats by Ficoll-Paque PLUS density (Cytiva) gradient centrifugation according to the manufacture’s instruction. CD56^+^ NK cells were then enriched by magnetic labelling using the human CD56^+^ NK cell Isolation Kit according to the manufacturer’s instruction (Miltenyi Biotec). NKG2C^-^NKG2A^+^, NKG2C^+^NKG2A^-^ and NKG2C^+^NKG2A^+^ NK cells were then sorted on a FACSAria Fusion (BD Bioscience). Sorted cells were stored frozen at −80 °C in 1x10^6^ viable cell per aliquots in 90% FCS + 10% DMSO (both: Thermo-Fisher).

### HLA-E stabilization and NKG2A^+^ NK cell inhibition experiments

For the HLA-E stabilization experiments, HLA-E*0101/0101-encoding Raji cells (German Collection of Microorganisms and Cell Cultures) were maintained in 90% RPMI 1640 + 10% heat-inactivated FCS (Thermo-Fisher). The cells were individually transfected with indicated concentrations of the *LMP-1* peptides (Peptides&Elephants) using the Pierce Protein Transfection Reagent according to the manufacturer’s instructions (Thermo-Fisher). After 16h, the cells were either fixed and analyzed for the HLA-E expression by flow-cytometry as described below, or washed once with Opti-MEM I Reduced Serum Medium (Gibco) and subsequently used in NKG2A^+^ NK cell inhibition experiments.

For the NKG2A^+^ NK cell inhibition experiments, sorted NKG2A^+^NKG2C^-^NK cells were quickly thawed at 37°C, washed, and pre-activated overnight in RPMI, 10% FCS, 1% L-glutamine (Thermo-Fisher), 10 ng/ml IL-12 (PeproTec) and 100 ng/ml IL-18 (Biozym Scientific) at 37°C. NKG2A^+^ NK cells were then harvested by centrifugation at 400xg for 5 minutes and washed once with Opti-MEM. The NK cells were then cultured together with peptide pulsed Raji cells. (Effector: Target ratio, E:T, 1:1) for 6 hours. After co-cultivation, the supernatant was removed, cleared by centrifugation (1000g, 5 minutes) and analyzed by IFNγ ELISA according to the manufacturer’s recommendations (Thermo Fisher).

### Cell proliferation assays

For the cell proliferation assays MHC-I-deficient K562-CR2, K562-CR2-HLA-E*0103/0103 or K562-CR2-HLA-E*0101/0101 target cells were established and maintained as described before (16). The cells were then always infected with the marmoset B-lymphoblastoid cell line B95-8 derived EBV-strain, which encodes for the *GGDPHLPTL* LMP-1 variant, (MOI=1) (16) for 3 days. Sorted NKG2C^-^NKG2A^+^, NKG2C^+^NKG2A^-^ and NKG2C^+^NKG2A^+^ NK cells were used as effector cells and were quickly thawed at 37°C, washed, and pre-activated overnight in RPMI, 10% FCS, 1% L-glutamine, 10 ng/ml IL-12 and 100 ng/ml IL-18 at 37°C. The NK cells were then harvested by centrifugation at 400xg for 5 minutes and washed once with Opti-MEM. The NK cells were then cultured together with EBV-infected K562-CR2, K562-CR2-HLA-E*0103/0103 or K562-CR2-HLA-E*0101/0101 cells (E:T, 1:1) in RPMI, 10% FCS, 1% L-glutamine for indicated time points. In some experiments, additional 300 µM of EBV *LMP-1* peptides or α-NKG2A blocking monoclonal antibodies (Monalizumab, 10 μg/mL, Innate Pharma) was added after 0, 2, and 4 days, respectively, to the co-culture. All cells were then harvested, fixed with the FIX & PERM Cell Fixation & Cell Permeabilization Kit (Thermo-Scientific) and analysed by flow-cytometry, as described below.

### Flow-cytometry

The following conjugated mouse anti-human mAB were used for flow-cytometry: PE-CD19 (4G7), BV421-CD56 (NCAM 16.2), BV510-NKG2A (131411), PE-CD71 (M-A712) (all: BD Biosciences), AF647-NKG2C (134591, R&D Systems), APC-HLA/E (3D12) (Biolegend). Dead cells were identified using 7-AAD, LIVE/DEAD Fixable Green or Near-IR Dead Cell Stain Kit (both: Thermo-Scientific). Flow-cytometry analysis was performed on a FACSCanto2 platform and FACSDiva Version 10.7.2 (BD).

### Statistical analysis

The Chi-square test and Fisher’s exact test were used to compare the distribution of the *KLRC2*, HLA-E and *LMP-1* variants. Outliers of the flow cytometry data were first identified using the ROUT method and then compared between the groups with the RM one-way ANOVA (with the Geisser-Greenhouse correction). A p-value < 0.05 was considered statistically significant. Statistical differences were assessed with GraphPad Prism 9.

## Results

### *LMP-1* peptide variants are associated with EBV^+^ Hodgkin and non-Hodgkin lymphomas

To analyze the role of EBV *LMP-1*-specific and HLA-E-mediated inhibitory NKG2A^+^ NK cell responses during EBV^+^ lymphomas, we first recruited 25 patients with EBV^+^HL and 38 patients with EBV^+^nHL (EBV^+^DLBCL: N=22, EBV^+^PTCL: N=16), 96 individuals with symptomatic EBV reactivations without any clinical evidence for EBV^+^ lymphomas (“symptomatic”) and additional 96 healthy EBV-IgG positive blood donors, which, in spite of a detectable EBV-viremia, had no symptoms (“asymptomatic”). Details of the study cohort are presented in **Table 1**.

**Table 1:** Characteristics of the study cohort.

Characteristic	Study Cohort				
	EBV^+^ Lymphoma			EBV Reactivations without Lymphoma	
	EBV^+^ HL N=25	EBV^+^ nHL N=38		Asymptomatic N=96 *	Symptomatic N=96
		EBV^+^DLBCL N=22	EBV^+^PTCL N=16		
Female (%)	N=7 (28%)	N=10 (45.5%)	N=6 (37.5%)	n/a	N=39 (49.6%)
Median Age (min-max)	48.8 (16–79)	65.4 (22–80)	61.4 (35–84)	n/a	37.8 (18 – 94)

DLBCL, diffuse large B-cell lymphoma, EBV, Epstein–Barr virus, HL, Hodgkin lymphoma, n/a: not available, nHL, non-Hodgkin lymphoma, PTCL, peripheral T cell lymphoma.

*The Asymptomatic cohort consists of healthy and anonymous blood donors.

All groups were tested for *LMP-1* peptide variants of the patients’ EBV-strains identified during the reactivation episodes. In overall, eleven different *LMP-1* peptide variants were identified (**Figure S1A**). Among these, the *GGDPHLPTL* (N=156, 61.2%) and *GSDPHLPTL* (N=22, 8.6%) variants were the most frequent, while the nine remaining variants only rarely occurred.

**Figure 1:**
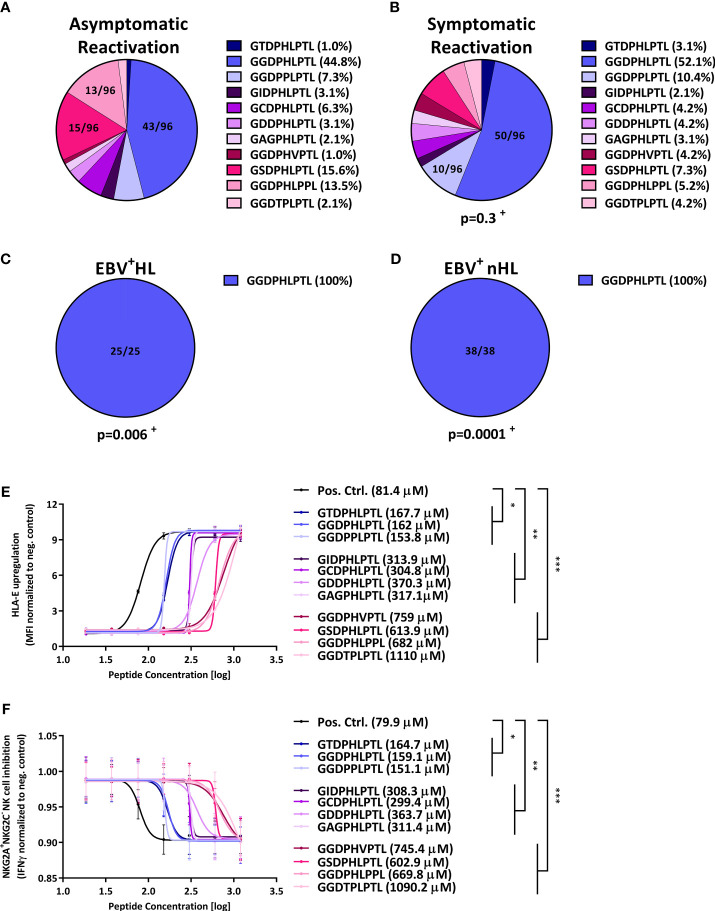
*LMP-1-*derived peptides are associated with the development of EBV^+^ lymphomas and inhibit NKG2A^+^ NK cells. **(A–D)** Distribution of *LMP-1* variants in patients with **(A)** asymptomatic reactivations (N=96), **(B)** symptomatic reactivations (N=96), **(C)** EBV^+^HL (N=25), **(D)** EBV^+^nHL (N=38). Fractions represent the relative frequency of the *LMP-1* peptide *GGDPHLPTL*, *GSDPHLPTL*, *GGDPHLPPL*, *GGDPPLPTL*, *GCDPHLPTL*, *GIDPHLPTL*, *GAGPHLPTL*, *GGDTPLPTL*, *GDDPHLPTL*, *GGDPHVPTL* and *GTDPHLPTL* variants. ^+^ The frequency of the *LMP-1* variants was compared to the asymptomatic cohort by the Chi^2^ Test. **(E, F)** HLA-E stabilisation assay. **(E)** HLA-E stabilisation assay: Raji cells were incubated with indicated concentrations of the positive control (*VMAPRTLFL*) or the *LMP-1* peptide-derived *GGDPHLPTL*, *GSDPHLPTL*, *GGDPHLPPL*, *GGDPPLPTL*, *GCDPHLPTL*, *GIDPHLPTL*, *GAGPHLPTL*, *GGDTPLPTL*, *GDDPHLPTL*, *GGDPHVPTL* and *GTDPHLPTL* variants. The HLA-E surface expression was then assessed after 16h of co-culture by flow-cytometry. Plots represent the mean ( ± SD) of three independent replicates. Each peptide was compared to the positive control using RM one-way ANOVA (with the Geisser-Greenhouse correction). **(F)** NKG2A^+^NKG2C^-^ NK cell inhibition assay: Raji cells were incubated with indicated concentrations of the positive control (*VMAPRTLFL*) or the *LMP-1* peptide-derived *GGDPHLPTL*, *GSDPHLPTL*, *GGDPHLPPL*, *GGDPPLPTL*, *GCDPHLPTL*, *GIDPHLPTL*, *GAGPHLPTL*, *GGDTPLPTL*, *GDDPHLPTL*, *GGDPHVPTL* and *GTDPHLPTL* variants and were then co-cultured with pre-activated NKG2A^+^NKG2C^-^ NK cells from 12 blood donors. Plots represent the mean ( ± SD) of 12 independent replicates. Each peptide was compared to the positive control using RM one-way ANOVA (with the Geisser-Greenhouse correction). p < 0.05 was considered significant. *p < 0.05; **p < 0.01; ***p < 0.001. EBV^+^HL, EBV^+^ Hodgkin lymphoma, EBV^+^nHL, EBV^+^ non-Hodgkin lymphoma, Pos. Ctrl., positive control.

We then compared the *LMP-1* peptide diversity between the grousps. While we observed a comparably high LMP-1 peptide diversity in patients with symptomatic and asymptomatic EBV infections (**Figures 1A, B**), the *LMP-1 GGDPHLPTL* peptide was the only variant we detected in EBV^+^HL and EBV^+^nHL patients (**Figures 1C, D**, **S1B-C**).

### The *LMP-1 GGDPHLPTL* peptide variant is associated with an efficient inhibition of NKG2A^+^ NK cells

Based on these results, we hypothesized that the *LMP-1 GGDPHLPTL* peptide is associated with an especially efficient HLA-E-mediated inhibition of NKG2A^+^ NK cells, which enables a potent immune evasion of *GGDPHLPTL-*encoding EBV strains.

Therefore, we transfected the EBV^+^ lymphoblast-like Raji cell line with varying concentration of each of the individual *LMP-1* peptide, found in the patients. As shown in **Figure 1E**, the transfection with the *GGDPHLPTL* peptide led, together with the overall less frequently occurring *GTDPHLPTL* and *GGDPPLPTL* peptides to a stable upregulation of HLA-E, as demonstrated by low EC50s, on the surface of Raji cells. To test, whether the stable *GGDPHLPTL*-mediated HLA-E upregulation is also associated with a potent inhibition of NKG2A^+^NKG2C^-^ NK cells, we co-cultured the *LMP-1* peptide pulsed Raji-cells together with pre-activated NKG2A^+^NKG2C^-^ NK cells, isolated from 12 healthy EBV-seropositive blood donors and subsequently measured the IFNγ concentration in the supernatant by ELISA. The *LMP-1 GGDPHLPTL* peptide led to a potent inhibition of NKG2A^+^NKG2C^-^ NK cells, as reflected by low IC50s (**Figure 1F**).

In contrast, the *LMP-1 GSDPHLPTL* and *GGDPHLPPL* variants, both frequently found in patients with symptomatic or asymptomatic EBV reactivations, but not in EBV^+^ lymphoma patients, showed only a low capacity to upregulate HLA-E on the surface of Raji cells (**Figure 1E**). Going along with this finding, both peptides led also to a poor inhibition of NKG2A^+^NKG2C^-^ NK cells (**Figure 1F**).

In summary, our data demonstrate that EBV^+^HL and EBV^+^nHL are hallmarked by EBV-strains that encode for the *LMP-1 GGDPHLPTL* variant, which induces a strong upregulation of cellular HLA-E and a potent inhibition of NKG2A^+^NKG2C^-^ NK cells.

### HLA-E variants are associated with symptomatic EBV reactivations and EBV^+^ lymphomas

**Figure 2:**
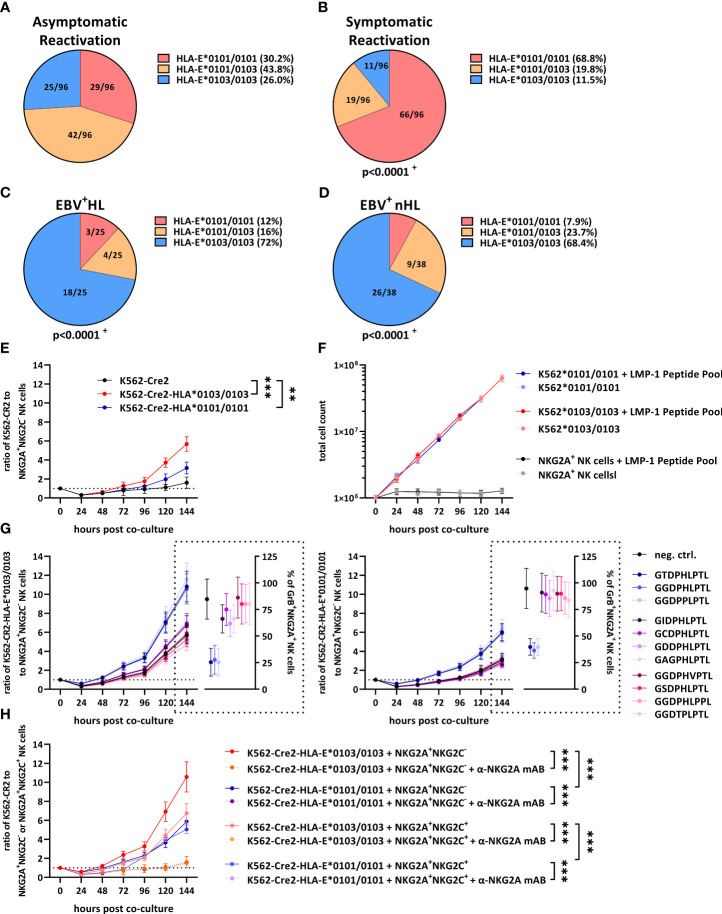
HLA-E variants are associated with the development of EBV^+^ lymphomas and inhibit NKG2A^+^ NK cells. **(A–D)** Distribution of HLA-E variants in patients with **(A)** asymptomatic reactivations (N=96), **(B)** symptomatic reactivations (N=96), **(C)** EBV^+^HL (N=25), **(D)** EBV^+^nHL (N=38). Fractions represent the relative frequency of HLA-E*0101/0101, HLA-E*0101/0103 and HLA-E*0103/0103. ^+^ The frequency of the HLA-E variants was compared to the asymptomatic cohort by the Chi^2^ Test. **(E–G)** Cell proliferation assays. **(E)** Enriched NKG2A^+^NKG2C^-^ NK cells were co-cultured with EBV-infected K562-CR2, K562-CR2-HLA-E*0103/0103 or K562-CR2-HLA-E*0101/0101 cells and were subsequently analyzed by flow-cytometry. **(F)** EBV-infected K562-CR2-HLA-E*0103/0103 or K562-CR2-HLA-E*0101/0101 cells or enriched NKG2A^+^NKG2C^-^ NK cells were co-cultured an LMP-1 peptide pool containing 300 µM of each of the *LMP-1* peptide-derived *GGDPHLPTL*, *GSDPHLPTL*, *GGDPHLPPL*, *GGDPPLPTL*, *GCDPHLPTL*, *GIDPHLPTL*, *GAGPHLPTL*, *GGDTPLPTL*, *GDDPHLPTL*, *GGDPHVPTL* and *GTDPHLPTL* variants. Viable EBV-infected K562-CR2-HLA-E*0103/0103 or K562-CR2-HLA-E*0101/0101 cells or enriched NKG2A^+^NKG2C^-^ NK cells was subsequently analysed by flow-cytometry. **(G)** Enriched NKG2A^+^NKG2C^-^ NK cells were co-cultured with EBV-infected K562-CR2, K562-CR2-HLA-E*0103/0103 or K562-CR2-HLA-E*0101/0101 cells and 300µM of the positive control (*VMAPRTLFL*) or the *LMP-1* derived *GGDPHLPTL*, *GSDPHLPTL*, *GGDPHLPPL*, *GGDPPLPTL*, *GCDPHLPTL*, *GIDPHLPTL*, *GAGPHLPTL*, *GGDTPLPTL*, *GDDPHLPTL*, *GGDPHVPTL* and *GTDPHLPTL* and were subsequently analysed by flow-cytometry. **(H)** Enriched NKG2A^+^NKG2C^-^ or NKG2A^+^NKG2C^+^ NK cells were co-cultured with EBV-infected K562-CR2, K562-CR2-HLA-E*0103/0103 or K562-CR2-HLA-E*0101/0101 cells and 300µM of the *GGDPHLPTL* variant and were subsequently analysed by flow-cytometry. For some experiments the α-NKG2A mAB 10µg/mL Monalizumab was added. **(E–G)** Plots represent the mean ( ± SD) of 12 independent replicates. RM one-way ANOVA (with the Geisser-Greenhouse correction) was used to compare the respective groups. p < 0.05 was considered significant. *p < 0.05; **p < 0.01; ***p < 0.001. EBV^+^HL, EBV^+^ Hodgkin lymphomas, EBV^+^nHL, EBV^+^ non-Hodgkin lymphomas, GrB, granzyme B, mAB, monoclonal antibody. Pos. Ctrl., positive control.

Beside the EBV *LMP-1*-encoded peptides, the HLA-E expression level on the cell surface of EBV-infected cells also depends on host-encoded HLA-E*0101/0103 variants (6). We therefore compared the HLA-E variants between the groups. As shown in **Figure 2A**, 30.2% of all patients with an asymptomatic EBV-infection encoded for the low-expressing HLA-E*0101/0101 variant, while 43.8% and 26% encoded for the heterozygous HLA-E*0101/0103 and the high-expressing HLA-E*0103/0103 allele, respectively. In contrast, the HLA-E*0101/0101 variant dominated in patients with symptomatic EBV infections (**Figure 2B**), while the HLA-E*0103 allele occurred only rarely, compared to patients with an asymptomatic EBV reactivation, (p<0.0001, OR: 5.1 (2.8-9.4), Fisher’s-test).

In EBV^+^HL (**Figures 2C**) and EBV^+^nHL (**Figures 2D**, **S2**) patients, the HLA-E*0103/0103 variant dominated, however, while patients encoding for the HLA-E*0101 allele were, in comparison to asymptomatic patients (EBV^+^HL: p<0.0001, OR: 7.3 (95% CI: 2.7-19.6); EBV^+^nHL: p<0.0001, OR: 6.1 (95% CI: 2.7-14), Fisher’s-test) and symptomatic patients (EBV^+^HL: p<0.0001, OR: 19.9 (95% CI: 6.8-58.3); EBV^+^nHL: p<0.0001, OR: 16.8 (95% CI: 6.6-42.4), Fisher’s-test) significantly underrepresented.

### The HLA-E*0103/0103 variant prevents the control of EBV-infected lymphoma cells

To test whether the HLA-E*0103/0103 variant is associated with an increased immune evasion, we cultured EBV B95-8-infected K562-CR2, K562-CR2-HLA-E*0103/0103 or K562-CR2-HLA-E*0101/0101 cells together with NKG2A^+^NKG2C^-^ NK cells, isolated from 12 healthy EBV-seropositive blood donors and monitored the growth of the cells *via* flow-cytometry. As shown in **Figure 2E**, K562-CR2-HLA-E*0103/0103 led to an efficient inhibition of NKG2A^+^NKG2C^-^ NK cells, resulting in a strong proliferation of EBV infected K562-CR2-HLA-E*0103/0103, compared to K562-CR2-HLA-E*0101/0101 cells or HLA-E-lacking K562-CR2 cells.

To test, whether the *LMP-1* peptide variants have an additional effect on the proliferation of EBV^+^ lymphoma cells, we first co-cultured the EBV-infected K562-CR2-HLA-E*0103/0103 or EBV-infected HLA-E*0101/0101 cells or NKG2A^+^NKG2C^-^ NK cells alone with an *LMP-1* peptide pool, containing all LMP-1 peptide variants. The LMP peptide pool alone did not alter the proliferation of the cells (**Figure 2F**). We then co-cultured the EBV-infected K562-CR2-HLA-E*0103/0103 or -HLA-E*0101/0101 cells with NKG2A^+^NKG2C^-^ NK cells and additional individual *LMP-1* peptides. As shown in **Figure 2G**, the addition of the *GGDPHLPTL* peptide led to a substantially increased proliferation of EBV-infected K562-CR2-HLA-E*0103/0103 and, to a lesser extent of K562-CR2-HLA-E*0101/0101 cells. We then also tested the activation of the NKG2A^+^NKG2C^-^ NK cells, as determined by Granzyme B-positive cells after six days of co-culture. The addition of the *GGDPHLPTL* peptide led to a substantially decreased percentage of Granzyme B-positive NKG2A^+^NKG2C^-^ NK cells (**Figures 2G**, **S3**)

In summary, our data demonstrate that a high HLA-E expression, induced by the high-expressing HLA-E*0103/0103 variant is not only associated with an increased risk for EBV^+^HL and EBV^+^nHL, but also with an efficient inhibition of NKG2A^+^ NK cells and an increased proliferation of EBV-infected tumour cells.

### Blocking of NKG2A activates NKG2A^+^NKG2C^-^ and NKG2A^+^NKG2C^+^ NK cells

The finding that HLA-E prevents the control of EBV-infected lymphoma cells *via* the stimulation of the inhibitory NKG2A receptor prompted us to test, whether the monoclonal antibody (mAB)-mediated blocking of NKG2A led to an effective control of EBV-infected tumor cells. Therefore, we cultured *GGDPHLPTL-*peptide pulsed EBV-infected K562-CR2-HLA-E*0103/0103 or HLA-E*0101/0101 cells together with NKG2A^+^NKG2C^-^ or NKG2A^+^NKG2C^+^ NK cells in the presence or absence of supplementary NKG2A-blocking mABs (Monalizumab). As shown in **Figure 2H**, the blocking of NKG2A generally led to a reduction of the EBV-infected K562-CR2 cell dissemination.

We then also compared the capacity of NKG2A^+^NKG2C^-^ or NKG2A^+^NKG2C^+^ NK cells to prevent the tumour cell dissemination. As shown in **Figure 2G**, NKG2A^+^NKG2C^+^ showed a significant reduction of EBV-infected K562-CR2-HLA-E*0103/0103 or HLA-E*0101/0101 cells, compared to NKG2A^+^NKG2C^-^ NK cells. In summary, our data demonstrate that NKG2A-blocking mABs can inhibit the EBV^+^ lymphoma cell dissemination, especially *via* activation of NKG2A^+^NKG2C^+^ NK cells.

### *KLRC2* deletion variants are associated with EBV^+^ lymphomas

The finding that NKG2A^+^NKG2C^+^, rather than NKG2A^+^NKG2C^-^ NK cells prevented the *in vitro* EBV^+^ lymphoma cell dissemination, prompted us to further analyse the role of NKG2C^+^ NK cells in the prevention of EBV^+^HL and EBV^+^nHL. The levels of NKG2A^-^NKG2C^+^ NK cells in the human host depend on the HCMV-serostatus, as well as on naturally occurring genetic homo- and heterozygous deletion variants in the NKG2C-receptor-encoding *KLRC2* gene (*KLRC2* ^wt/del^ *, KLRC2* ^del/del^).

**Figure 3:**
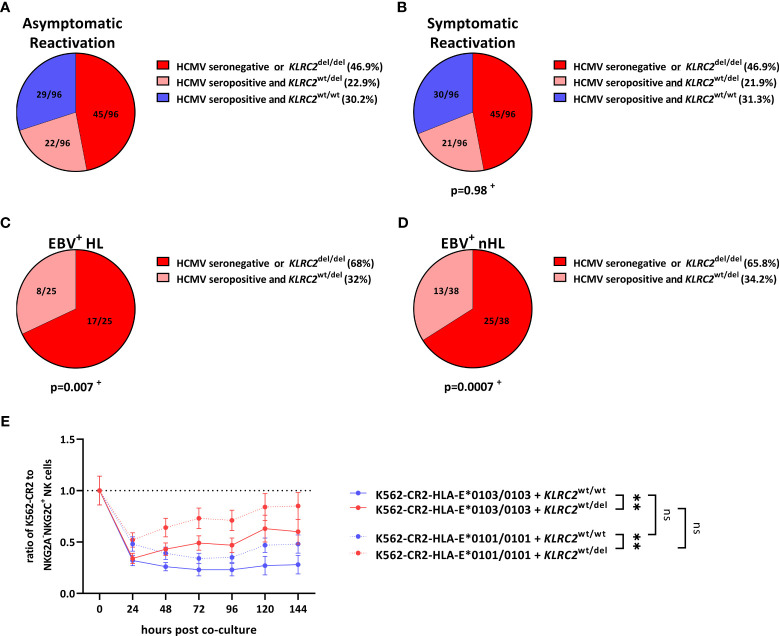
*KLRC2* variants are associated with the development of EBV^+^ lymphomas. **(A–D)** Distribution of *KLRC2* variants in HCMV-seropositive and HCMV-seronegative individuals with **(A)** asymptomatic reactivations (N=96), **(B)** symptomatic reactivations (N=96), **(C)** EBV^+^HL (N=25), **(D)** EBV^+^nHL (N=38). Fractions represent the relative frequency of the *KLRC2* variants in HCMV-seropositive and HCMV-seronegative individuals. ^+^ The frequency of the *KLRC2* variants was compared to the asymptomatic cohort by the Chi^2^ Test. **(E)** Cell proliferation assays. Enriched NKG2A^-^NKG2C^+^ NK cells from 12 healthy blood donors encoding for the *KLRC2* ^wt/wt^ (N=6) and *KLRC2* ^wt/del^ (N=6) variant were co-cultured with EBV-infected K562-CR2-HLA-E*0103/0103 or K562-CR2-HLA-E*0101/0101 cells, pulsed with the *LMP-1 GGDPHLPTL* variant and were subsequently analysed by flow-cytometry. Plots represent the mean ( ± SD) of 6 independent replicates. RM one-way ANOVA (with the Geisser-Greenhouse correction) was used to compare the respective groups. p < 0.05 was considered significant. **p < 0.01. del, deletion, EBV^+^HL, EBV^+^ Hodgkin lymphomas, EBV^+^nHL, EBV^+^ non-Hodgkin lymphomas, ns, not significant, wt, wild type.

We therefore tested all EBV^+^ lymphoma patients and the respective controls for their HCMV-serostatus and *KLRC2* variants. As shown in **Figures 3A, B**, 30.2% of asymptomatic and 31.3% of symptomatic EBV-infected patients were HCMV-seropositive and encoded for the *KLRC2* ^wt/wt^, reflecting an overall strong NKG2C^+^ NK cell response. However, the same combination was completely absent in EBV^+^HL and EBV^+^nHL patients (**Figures 3C, D**, **S4**).

### NKG2C^wt/wt^ NK cells efficiently control the proliferation of EBV^+^ lymphoma cells

To further evaluate, whether the *KLRC2* ^wt/del^ allelic configurations are associated with the development of EBV^+^ lymphomas, we co-cultured EBV-infected K562-CR2-HLA-E*0103/0103 or K562-CR2-HLA-E*0101/0101 cells together with NKG2A^-^NKG2C^+^ NK cells, isolated from 12 HCMV-seropositive donors, encoding for the *KLRC2* ^wt/wt^ (N=6) and *KLRC2* ^wt/del^ (N=6) variants. As shown in **Figure 3E**, NKG2C^wt/wt^ inhibited the dissemination of EBV^+^ lymphoma cells significant more efficient than NKG2C^wt/del^ NK cells. In summary, our data thus demonstrate that NKG2C^wt/wt^ NK cells can efficiently control the proliferation of EBV^+^ lymphoma cells.

## Discussion

In the present study, we show that the development of the EBV-associated and life-threatening malignant diseases EBV^+^HL and EBV^+^nHL is associated with distinct virus- and host-associated factors which affect NKG2C^+^ and NKG2A^+^ NK cell responses.

EBV^+^HL, EBV^+^PTCL and EBV^+^DLBCL are malign diseases caused by EBV and are associated with a poor overall survival (17–19). As more than 90% of the adult population carry EBV (20) and undergo sporadic EBV reactivations, the question remains why some of the persons with reactivating EBV progress towards EBV^+^ lymphomas, while in most others, the reactivation episodes are efficiently controlled. We now reveal that the development of EBV^+^HL, EBV^+^PTCL and EBV^+^DLBCL is significantly associated with variations in EBV-specific, HLA-E-restricted immune responses. We uncovered, that the interplay of three factors, a specific viral *LMP-1* peptide variant of the infecting EBV-strain, the high-expressing host HLA-E*0103/0103 genotype and the absence of a potent host NKG2C^+^ NK cell response, is associated with a particularly high-risk for these EBV^+^ lymphomas.

EBV^+^HL and EBV^+^nHL are generally characterized either by the EBV latency II or III gene expression profile, respectively, which are hallmarked by a high expression of EBV *LMP-1* (21). The circulating EBV-strains, detected in patients with symptomatic or asymptomatic EBV reactivations in the present study showed a high *LMP-1*-derived peptide diversity, as previously described for patients with symptomatic primary EBV-infections and symptomatic EBV reactivations (16). However, in patients, in whom the EBV reactivation progressed to EBV^+^HL and EBV^+^nHL, only EBV strains, which encoded for the *LMP-1 GGDPHLPTL* peptide variant were found. This is in agreement with earlier data showing that EBV-strains, encoding for this *LMP-1* peptide variant were also a risk factor for the development of EBV^+^ post-transplant lymphoproliferative disorders (EBV^+^PTLD) (16).

In search for the functional background of these findings, we could show that the *GGDPHLPTL* peptide results in an efficient upregulation of HLA-E and an efficient inhibition of the NKG2A^+^ NK cell-mediated secretion of IFNγ. Earlier studies identified a distinct NKG2A^+^ NK cell subset in the tonsils of EBV-carriers, which produces large amounts of IFNγ that prevents malignant B cell transformation (22). Together, these and our data provide evidence that NKG2A^+^ NK cells can, to some extent, prevent the EBV-induced transformation and that the EBV-mediated inhibition of NKG2A^+^ NK cells subsequently increases the risk to develop EBV^+^HL and EBV^+^nHL.

Beside the polymorphic EBV-encoded *LMP-1* peptide variants, also the host-encoded HLA-E*0101/0103 genotypes modulate the expression of HLA-E. In the present study we could demonstrate that the homozygous HLA-E*0103/0103 variant is significantly overrepresented in individuals who did develop EBV^+^HL, EBV^+^DLBC and EBV^+^PTCL. The HLA-E*0103/0103 genotype was previously shown to provide a more efficient assembly with β2-microglobulin and a faster ER egress, compared to the HLA-E*0101/0101 genotype, resulting in a high-level HLA-E expression on the surface of EBV-infected cells (6). In addition, HRS cells are characterized by a high level of HLA-E expressing cells and increased HLA-E expression was further associated with an advanced clinical stage of cHL (23, 24). In agreement with our findings, a recent study identified the HLA-E*0101 variant as a protective factor for EBV^+^ cHL (25) and EBV^+^PTLD (16), which further confirms that the HLA-E genotype impacts the development of EBV-associated lymphomas.

Our study revealed that the high-expressing HLA-E*0103/0103 variant does not only result in a potent inhibition of NKG2A^+^ NK cells, but also in the strong activation of NKG2A^+^NKG2C^+^ NK cells. A genetic-association study recently demonstrated that the *KLRC2* ^del/del^ genotype is significantly overrepresented and even associated with a reduced progression-free survival in B-nHL patients (26). High NKG2C^+^ NK cell levels in a patient depend, however, not only on the host *KLRC2* ^wt/wt^ genotype, but also on previous HCMV infections, as shown by a positive HCMV-serostatus. We could demonstrate that a combination of these two factors, both leading to strong NKG2C^+^ NK cell responses, was completely absent in the EBV^+^HL and EBV^+^DLBCL patients of our cohort. In contrast, low level or even lacking NKG2C^+^ NK cell responses, reflected by the combination of a negative HCMV-status and *KLRC2* deletion variants, were highly associated with the development of EBV^+^ lymphomas. As the functional background of these findings, we could reveal that NKG2C^+^ NK cells efficiently control the proliferation of EBV-infected lymphoma cells. NKG2A^-^NKG2C^+^ NK cells are highly cytotoxic and additionally secrete pro-inflammatory cytokines. Interestingly, leukaemia and lymphoma patients, undergoing hematopoietic cell transplantation had a reduced relapse-risk when experiencing HCMV reactivations, and it was hypothesized that NKG2C^+^ NK cells expanding in response to HCMV may contribute to protection against relapses (27, 28).

In our study, we could show that also NKG2A^+^NKG2C^+^ NK cells efficiently control the proliferation of EBV-infected tumor cells, when they were treated with NKG2A-blocking monoclonal antibodies (Monalizumab). These findings are of special interest, as efficient therapies for EBV^+^HL and EBV^+^DLBC are still scarce and Monalizumab is a drug so far already under investigation for the treatment of distinct gynecological, lung and colorectal cancers (29, 30). Further extended studies are required to evaluate the potential of NKG2A-blocking antibodies for the treatment of EBV^+^HL, EBV^+^DLBC and EBV^+^PTCL.

In summary, we have identified that the progression to EBV^+^HL and EBV^+^nHL in the individual patients was highly associated with a potent inhibitory NKG2A/*LMP-1*/HLA-E pathway, but absent pro-inflammatory NKG2C^+^ NK cell responses. Further extended studies are, however, needed to evaluate whether the analysis of individual and combined variations in the HLA-E-restricted immune response may serve as prognostic markers for malignant EBV-associated diseases and to assess whether drug-induced blocking of NKG2A may provide a therapeutic option for EBV^+^ lymphomas.

## Data availability statement

The original contributions presented in the study are included in the article/**Supplementary Material**. Further inquiries can be directed to the corresponding author.

## Ethics statement

The studies involving human participants were reviewed and approved by Ethics committee of the Medical University of Vienna. Written informed consent for participation was not required for this study in accordance with the national legislation and the institutional requirements.

## Author contributions

Conceptualization: HV and EP-S; Data curation: HV, SB, PF, LK; Formal analysis: HV, Funding acquisition: EP-S; Investigation: HV and EP-S; Methodology: HV; Project administration: EP-S; Resources: PS, SL, AP, RS, JC; Supervision: EP-S; Validation: HV; Visualization: HV; Writing-original draft: HV and EP-S. All authors contributed to the article and approved the submitted version.
