# Supplementary material for: Inhibitory NKG2A+ and absent activating NKG2C+ NK cell responses are associated with the development of EBV+ lymphomas
Source: Front Immunol. 2023 Jun 22;14:1183788. doi: 10.3389/fimmu.2023.1183788 (PMC10324562; doi:10.3389/fimmu.2023.1183788)
Supplement: Supplementary file 1 [file DataSheet_1.pdf]

Supplementary Material

Inhibitory NKG2A^{+} and absent activating NKG2C^{+} NK cell responses are associated with the development of EBV+ lymphomas

Hannes Vietzen [1], Philipp B. Staber [2], Sarah M. Berger [1], Philippe L. Furlano [1], Laura M. Kühner [1], Simone Lubowitzki [2], Alexander Pichler [2], Robert Strassl [3], Jan J. Cornelissen [4] and Elisabeth Puchhammer-Stöckl [1]

*** Correspondence:** Hannes Vietzen: hannes.vietzen@meduniwien.ac.at

Figure S1

A

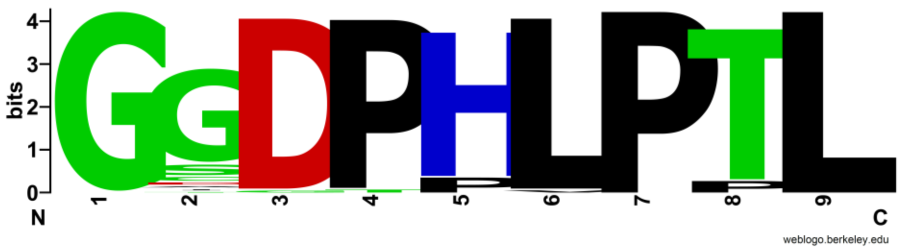

B

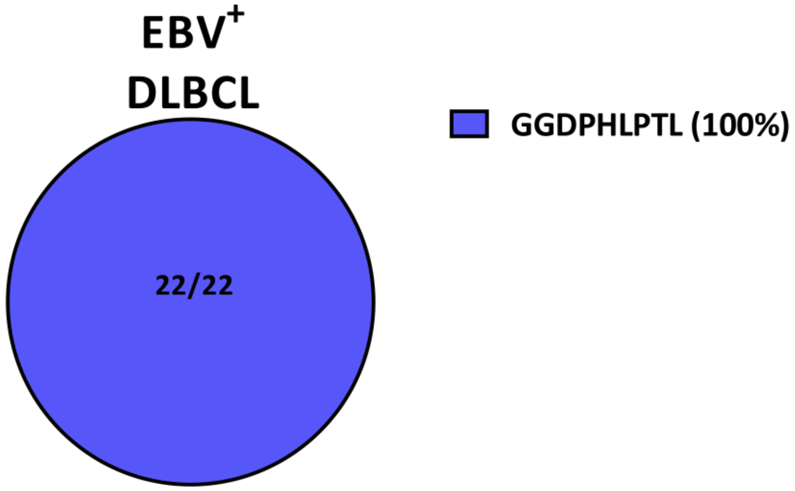

C

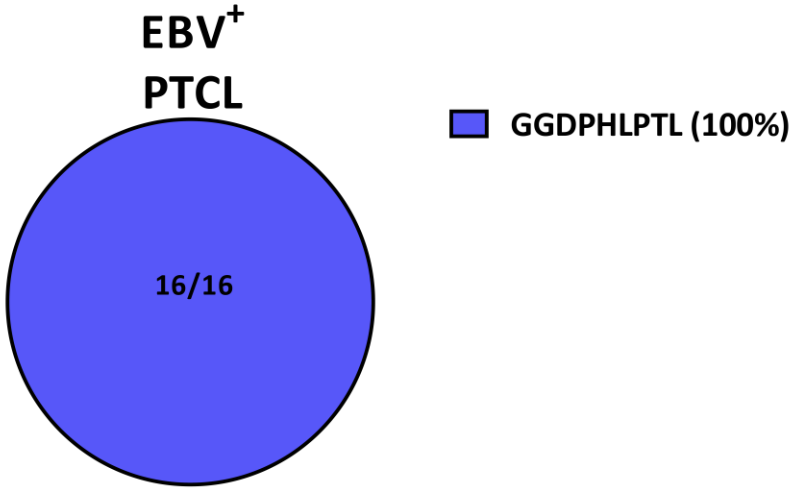

Figure S1: Sequence logo alignment and relative frequency of *LMP-1* peptide variants in EBV^{+}DLBCL and EBV^{+}PTCL patients. (A) Sequence logo alignment of the relative frequency of *LMP-1* peptide variants from *N*=255 partially sequenced *LMP-1* genes from patients with an asymptomatic EBV reactivation (N=96), symptomatic EBV reactivation (N=96), EBV^{+} HL (N=25) and EBV^{+} nHL (N=28). Sequence logos were created with the web-tool of the University of California, (https://weblogo.berkeley.edu/). (B-C) Distribution of *LMP-1* variants in patients with (B) EBV^{+}DLBCL (N=22) and (C) EBV^{+}PTCL (N=16). Fractions represent the relative frequency of the *LMP-1* peptide *GGDPHLPTL*, *GSDPHLPTL*, *GGDPHLPPL*, *GGDPPLPTL*, *GCDPHLPTL*, *GIDPHLPTL*, *GAGPHLPTL*, *GGDTPLPTL*, *GDDPHLPTL*, *GGDPHVPTL* and *GTDPHLPTL* variants. **DLBCL:** Diffuse large B cell lymphoma, **EBV:** Epstein–Barr virus, **PTCL:** Peripheral T cell lymphoma.

Figure S2

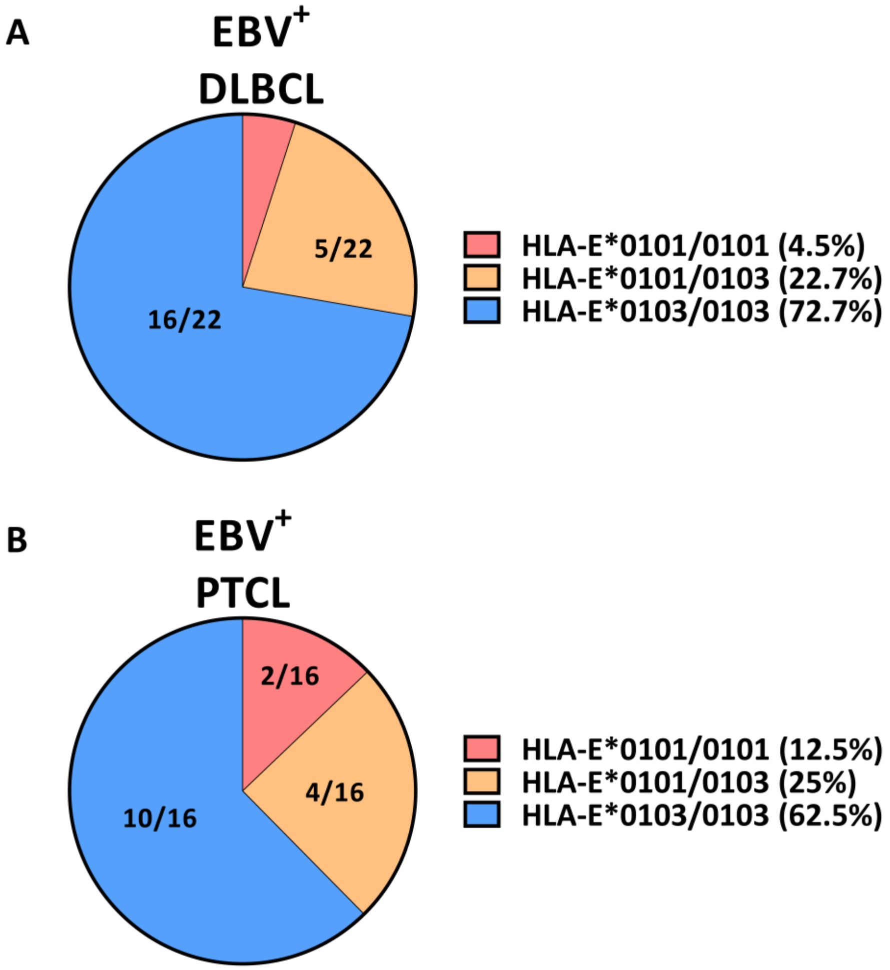

Figure S2: HLA-E variants are associated with the development of EBV^{+}DLBCL and EBV^{+}PTCL. (A-B) Distribution of HLA-E variants in patients with (A) EBV^{+}DLBCL (N=22) and (B) EBV^{+}PTCL (N=16). Fractions represent the relative frequency of HLA-E*0101/0101, HLA-E*0101/0103 and HLA-E*0103/0103. **DLBCL:** Diffuse large B cell lymphoma, **EBV:** Epstein–Barr virus, **PTCL:** Peripheral T cell lymphoma.

Figure S3

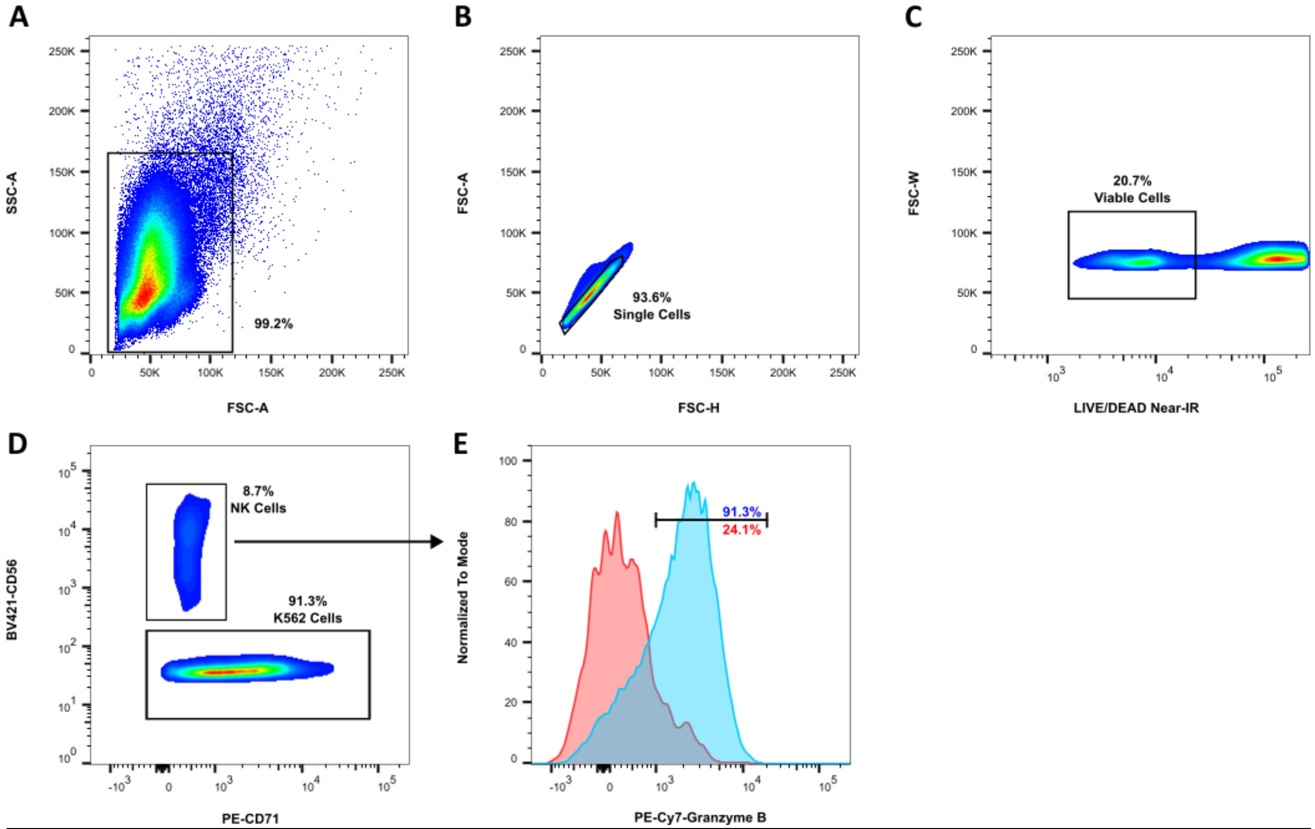

Figure S3: Proliferation Assay: NKG2A$^+$CD56$^+$ NK cells from one healthy blood donor were cultured in the presence of K562-CR2-HLA-E*0103/0103 cells and 300 µM of EBV *LMP-1 GGDPHLPTL* peptide for 6 days. The frequency of (A-D) viable CD56$^+$ NK cells or K562-CR2-HLA-E*0103/0103 cells was assessed by flow-cytometry. (E) The percentage of activated (gGranzyme B$^+$) CD56$^+$ NK cells (red) was assessed in comparison to the negative control, *e.g*. NKG2A$^+$CD56$^+$ NK cells, cultured together with K562-CR2-HLA-E*0103/0103 cells, but without LMP-1 peptides. One representative example of one blood donor is shown.

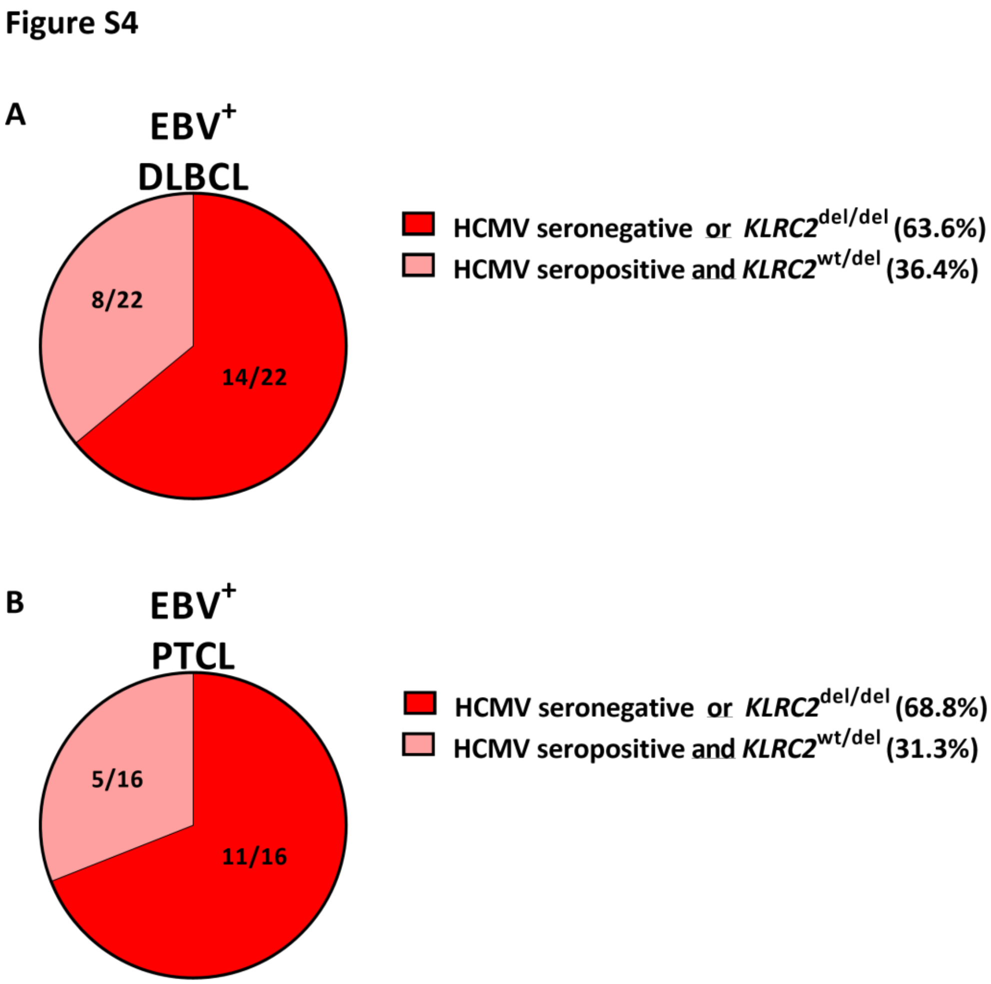

Figure S4: *KLRC2* variants are associated with the development of EBV^+DLBCL and EBV^+ PTCL. (A-B) Distribution of *KLRC2* variants in HCMV-seropositive and HCMV-seronegative individuals with (A) EBV^+DLBCL (N=22) and (B) EBV^+PTCL (N=16). Fractions represent the relative frequency of in *KLRC2* variants in HCMV-seropositive and HCMV-seronegative individuals. **DLBCL:** Diffuse large B cell lymphoma, **EBV:** Epstein–Barr virus, **PTCL:** Peripheral T cell lymphoma.
